# Exploring the Dietary Patterns of Young New Zealand Women and Associations with BMI and Body Fat

**DOI:** 10.3390/nu8080450

**Published:** 2016-07-26

**Authors:** Jenna K. Schrijvers, Sarah A. McNaughton, Kathryn L. Beck, Rozanne Kruger

**Affiliations:** 1 School of Food and Nutrition, Massey University, Auckland 0745, New Zealand; jenna.schrijvers@hotmail.com (J.K.S.); k.l.beck@massey.ac.nz (K.L.B.); 2 Institute for Physical Activity and Nutrition, School of Exercise and Nutrition Services, Deakin University, Melbourne 3125, Australia; sarah.mcnaughton@deakin.edu.au

**Keywords:** dietary assessment, dietary patterns, premenopausal women, food frequency questionnaire

## Abstract

Examining dietary patterns provides an alternative approach to investigating dietary behaviors related to excess adiposity. The study aim was to investigate dietary patterns and body composition profiles of New Zealand European (NZE) women, participating in the women’s EXPLORE (Examining the Predictors Linking Obesity Related Elements) study. Post-menarche, pre-menopausal NZE women (16–45 years) (*n* = 231) completed a validated 220-item, self-administrated, semi-quantitative food frequency questionnaire. Body mass index (BMI) was calculated using measured height (cm) and weight (kg); body fat percentage (BF%) was measured using air displacement plethysmography (BodPod). Dietary patterns were identified using principal component factor analysis. Associations between dietary patterns, age, BMI and BF% were investigated. Four dietary patterns were identified: snacking; energy-dense meat; fruit and vegetable; healthy, which explained 6.9%, 6.8%, 5.6% and 4.8% of food intake variation, respectively. Age (*p* = 0.012) and BMI (*p* = 0.016) were positively associated with the “energy-dense meat” pattern. BF% (*p* = 0.016) was positively associated with the “energy-dense meat” pattern after adjusting for energy intake. The women following the identified dietary patterns had carbohydrate intakes below and saturated fat intakes above recommended guidelines. Dietary patterns in NZE women explain only some variations in body composition. Further research should examine other potential factors including physical activity and socioeconomic status.

## 1. Introduction

The prevalence of obesity has significantly risen both in New Zealand (NZ) and worldwide, with 32% of all females in NZ classified as obese [1]. It is well-established that obesity is a major risk factor for many health conditions including but not limited to cardiovascular disease, type 2 diabetes, hypertension, insulin resistance, dyslipidemia, osteoarthritis, sleep apnoea, psychological and social problems, and some cancers [2].

The most widely used indicator to quickly identify overweight or obesity is the Body Mass Index (BMI), determined by weight/height^2^ (kg/m^2^) [2,3]. In NZ, overweight and obesity are defined as a BMI between 25 kg/m^2^ and 29.9 kg/m^2^, and ≥30 kg/m^2^, respectively [2,4]. Although routinely used in epidemiological studies and by health professionals, BMI is an imperfect measure of body fatness [5]. It speculates that at any given height, a higher weight correlates to a larger body fat percentage (BF%) and consequently a higher risk of morbidity and mortality [6]. Adiposity may be overestimated in individuals who have a high lean muscle mass as they are incorrectly classed as overweight (e.g., athletes), whereas underestimation may occur for others who have a normal BMI but excess body fat (>30%) (e.g., premenopausal women) [7]. It is estimated that more than half of individuals are misclassified when using BMI categorization [2]. Air displacement plethysmography (ADP) is the emerging gold standard to accurately measure BF% [8]. Reviewing both BMI and BF% allows accurate estimation of body composition, and in combination with lifestyle and dietary factors, allows the risk of chronic disease to be assessed and the tailoring of appropriate dietary advice.

Both BMI and body composition (i.e., ratio of fat mass to lean muscle mass) are influenced by lifestyle and dietary factors, such as the types of foods habitually consumed. Younger, premenopausal women are particularly at risk of higher BF% due to poor lifestyle (e.g., physical inactivity) and eating (e.g., dieting, fast foods) behaviors [9]. Traditionally nutritional epidemiology has focused on the effects of individual foods and nutrients on health outcomes. Although many studies have investigated the effects of specific macronutrients on body composition, dietary patterns have received less attention [10]. As foods and nutrients are consumed in combination, dietary patterns have recently emerged as an effective way to assess diet-disease relationships [11].

Specific dietary patterns such as a “Western” dietary pattern have been associated with weight gain and an increased risk of cardiovascular disease, type 2 diabetes, and cancer [12]. This pattern is characterized by a habitual intake of refined grains, breads and cereals, red and processed meats, fast-food, sugar-sweetened beverages (SSB), sweets and desserts [13]. In comparison, a diet composed of fruit, vegetables, whole grain bread and cereals, lean unprocessed meats, poultry, fish and low fat dairy products has been found to be independently associated with a higher dietary adequacy, lower BMI, and lower waist circumference, thus reducing the risk of consequent chronic disease [13,14]. Practicing these dietary patterns may also determine body composition, particularly body fatness, which may in turn influence morbidity and mortality risk factors. No studies to date have investigated associations between BF% and dietary patterns in premenopausal women.

Another important aspect of dietary patterns is their macronutrient composition which provides further information regarding the make-up of the dietary pattern. Studies have found that when the BF% of women increased, the percentage of fat derived from their diet also increased (35%) and the carbohydrate content decreased (46%) compared to lean individuals (29% and 53%, respectively) [15]. However, studies have not concurrently examined dietary patterns and the macronutrient profile of the diet in relation to BF%. Consideration of dietary patterns and body composition may enable the tailoring of dietary interventions appropriate for women with different body fat profiles to improve their morbidity and mortality outcomes. The aims of this study were to identify the dietary patterns of New Zealand European women (NZE) aged 16–45 years and examine their associations with BMI, body fat and macronutrient profile intakes.

## 2. Materials and Methods

### 2.1. EXPLORE Study Design

This study was a sub-study of the women’s EXPLORE (Examining Predictors Linking Obesity Related Elements) study [16]. The EXPLORE study investigated how body weight and body fat profiles are related to the risk for chronic disease in premenopausal women as well as investigating how diet and physical activity patterns may impact these profiles and how they may affect miRNA associated with body fat usage and storage [16]. The study protocol was reviewed and approved by the Massey University Ethics Committee: Southern A, Application 13/13. Further information regarding the EXPLORE study protocol can be found elsewhere [16]. The study took place in the Human Nutrition Research Unit (HNRU) at Massey University’s Albany Campus. Recruitment took place on-site at the HNRU, and throughout Auckland.

### 2.2. Study Participants and Procedures

Participants included 233 post-menarche, pre-menopausal NZE women, aged 16 to 45 years who were not pregnant or breastfeeding, were free from chronic disease/illness and not seeking to actively lose or gain weight. Participants who registered their interest were provided with an information sheet and completed an online health screening and demographics questionnaire to determine eligibility. If the inclusion criteria were met, participants were invited to participate in the screening phase (Phase 1). The study entailed two phases: screening (phase 1) and data collection (phase 2).

#### 2.2.1. Phase 1 (Screening)

Screening took place at the HNRU or off-site at a community venue convenient and appropriate for recruited participants. Simple anthropometric measurements including height, weight, and body fat composition were obtained for the body composition screening. Height was measured using a portable stadiometer. Weight and BF% were obtained using the bioelectrical impedance analysis (BIA) machine (Biospace, Inbody 230, Cerritos, CA, USA). The BIA results were not shown to the participants to ensure a blinded study. Participant’s BMI (calculated using height and weight) and BF% results were used for preliminary categorization into body composition groups to ensure a range of BMI and BF% (normal to obese) as required for the EXPLORE study [16]. Ineligible participants were identified and excluded from the study.

#### 2.2.2. Phase 2 (Data Collection)

Participants who fit the body composition grouping criteria for the EXPLORE study in phase 1 visited the HNRU for approximately two hours, arriving between 7 a.m. and 9:45 a.m. Participants completed consent forms, a health questionnaire, and a battery of tests [16]. For the purposes of this sub-study, the following data was collected: anthropometric measures including height (cm), weight (kg), and a full body composition analysis conducted via air displacement plethysmography (ADP) (2007A, Life Measurement Inc., Concord, CA, USA, using software V4.2+ as supplied by the manufacturer) using the thoracic gas volume method in the BodPod [17,18]. Standard procedures for body composition assessment in the BodPod include no food, drink or physical activity two hours prior to assessment, recommended clothing (swimwear and caps), removal of all metal objects, and an empty bladder. Noseclips and breathing tubes were provided.

Participants also completed a validated 220-item, self-administered, semi-quantitative NZ women’s food frequency questionnaire (NZWFFQ) to assess dietary intake over the previous month [19]. The NZWFFQ has good relative validity when ranking individuals in relation to their macro and micronutrient intakes [19]. The women responded on a nine point scale (ranging from never to 4+ times a day) their frequency of eating each particular food grouping based on an average standard portion size. All NZWFFQ frequencies were entered into FoodWorks 7 (FoodWorks Professional, 2013) dietary analysis database using the NZ Food Composition Database (NZ, FOODfiles 2010) developed by New Zealand Plant and Food Research to determine energy, fiber and macronutrient intakes (carbohydrate, protein, total fat, saturated fat). An average of the range of frequencies reported was used (e.g., 2–3x/day was averaged to 2.5x/day). The entered data on monthly and weekly intakes were converted into daily equivalent frequencies as follows: never (0.00), <1x/month (0.03), 1–3x/month (0.06), 1x/week (0.14), 2–3x/week (0.36), 4–6x/week (0.78), once/day (1.00), 2–3x/day (2.50), 4+ x/day (4.00).

### 2.3. Data Analysis

The Goldberg equation was performed to identify participants who misreported their energy intake in the NZWFFQ in relation to their energy requirements. Schofield’s equations were used to determine participant basal metabolic rates (BMR) (kilojoules per day). A physical activity level (PAL) cut off point of 1.55 was used for all participants as this was determined to be a conservative value which represented seated work and very little strenuous leisure activity [20]. To derive the cut offs for evaluation of misreporting of energy intakes the SD_min_ and SD_max_ values of −2 and +2 were used for 95% lower and upper confidence intervals, respectively. The revised factors outlined in Black [20] were used in this analysis. Literature reports large bias in energy reporting of overweight and obese individuals [21]. To ensure accuracy, participants who were identified as misreporters were excluded in the macronutrient analysis but were included in the dietary pattern analysis. 

Dietary patterns (using SPSS 21.0 for Windows (SPSS Inc., Chicago, IL, USA)) were derived using exploratory factor analysis with factor loadings extracted using principal component analysis, varimax rotation and eigenvalues greater than 1. All participants were included in the factor analysis. Each of the food items were arranged into one of 56 food groups (Supplementary Materials Table S1) which were entered into the principal component analysis as daily equivalent frequencies (as described above). The number of food groups created was based on the recommendation by Kass and Tinsley [22] to allocate a minimum of 5–10 participants per food group. The Kaiser-Meyer-Olkin (KMO) measure of sampling adequacy and the Bartlett’s test of sphericity were used to assess the adequacy of correlation matrices within the data set. Scree plots using the break-even point, eigenvalues of at least 1.75 and the factors themselves were examined to identify which factors best explained the data. Cronbach’s α coefficients were used to assess inter-item reliability. Food items within each pattern that indicated that they were skewing the reliability and would increase the scale when taken out were removed from the pattern (for example, pattern 4’s total Cronbach α coefficient increased from 0.252 to 0.431 when oil and oil-based dressings, fats and soy milk were removed from the pattern). The factor analysis was re-run without these items to ensure that the removal increased reliability without significantly influencing the dietary patterns [23].

Simplified dietary pattern scores were calculated by summing the daily equivalent frequencies for each food group that loaded highly on each of the patterns. Food and beverages that loaded highly (≥0.2) were positively associated with the factor and largely contributed to the identified dietary pattern [24]. A negative loading indicated that the group was inversely associated with the factor and were not considered as contributing to the dietary pattern. Food groups which cross-loaded on multiple factors were removed from the dietary pattern with the lowest factor loading. Tertiles for each of the patterns were then determined. A one-way ANOVA was performed to compare the mean age, BMI, BF%, energy, fiber, and macronutrient intake within tertiles of each dietary pattern. Post hoc tests of Tukey and Gabriel were used to determine which tertiles were significantly different from one another. Energy intake and age adjustments using ANCOVA were made when comparing variables across the four dietary pattern tertiles. A significant interaction existed for the “fruit and vegetable” dietary pattern tertiles, and energy intake with respect to age (*p* = 0.03). For women in tertile 3 as age increased, energy intake decreased. For tertiles 1 and 2, as age increased, energy intake also increased. No other significant interactions were observed.

## 3. Results

### 3.1. Participants

A total of 473 NZE women were recruited of which 233 women met the inclusion criteria and were invited to complete the study protocol. Two women were excluded from this sub-study as they did not complete the NZWFFQ. Twenty four women were identified as misreporters using the Goldberg equation (19 under-reporters, 5 over-reporters), based on energy intakes relative to their energy requirements [20].

### 3.2. Participant Characteristics

The NZE women had an average age of 31.9 years, a median BMI of 24 kg/m^2^ (healthy weight) and a mean BF% of 32.6% (Table 1). The majority of the women were employed (*n* = 191, 82.7%), non-smokers (*n* = 221, 95.7%) and did not have any children (*n* = 156, 67.5%).

### 3.3. Dietary Patterns

Dietary patterns analysis was conducted from the NZWFFQ data obtained from the eligible NZE population. All NZE women were included in the factor analysis as it is thought that including misreporters in the analysis of dietary patterns does not significantly bias the data outputs [26]. Principal components factor analysis identified four unique dietary patterns (Table 2).

The four patterns were named based on food groups that loaded highly according to similar food and nutritional characteristics. Pattern 1 was the “snacking” pattern and was characterized by high loadings on peanut butter, margarine, sweet spreads, cakes and biscuits, and sweet and savory snack foods e.g., chocolate and potato chips; and negative loadings on green vegetables, red meats, and egg and egg dishes. Pattern 2 (“energy-dense meat” pattern) was characterized by high loadings on red, white, and processed meats, puddings and crumbed and deep fried foods, e.g., crumbed and battered fish; and negative loadings on high calcium milk, yoghurt, and soy products. The third pattern was named the “fruit and vegetable” pattern and was characterized by high loadings on all fruit and vegetable food groups; and negative loadings on coffee. The fourth pattern identified was the “healthy” pattern which was characterized by high loadings on whole grains, fish and seafood, legumes, nuts and seeds; and negative loadings on brown breads and sweetened cereals.

Women with higher scores on the “snacking” pattern were older than those with lower scores on the “snacking” pattern (33.9 ± 7.9 years vs. 31.9 ± 8.7 years, *p* = 0.026). This association between the “snacking” pattern remained after adjusting for energy intake (*p* = 0.011). There were no significant differences between BMI and BF% across the “snacking” dietary pattern tertiles, even after adjusting for age and energy intake. Women with higher scores in the “energy-dense meat” pattern were younger (30.0 ± 8.3 years) compared to those less likely to follow the “energy-dense meat” dietary pattern (32.8 ± 8.2 years, *p* = 0.048). This association remained after adjusting for energy intake (*p* = 0.012). Women with higher scores on the “energy-dense meat” pattern had a higher BMI (26.4 ± 6.7 kg/m^2^) compared to those with lower scores on the “energy-dense meat” pattern (24.6 ± 4.2 kg/m^2^, *p* = 0.036). This association remained after adjusting for age (*p* = 0.016), but was no longer significant after adjusting for energy intake. A significant association was also observed between the “energy-dense meat” pattern and BF% after adjusting for age (*p* = 0.016), but not for energy intake. No significant associations were seen for any of the variables with the “fruit and vegetable” and the “healthy” patterns, even after appropriate energy intake and age adjustments (Table 3).

### 3.4. Macronutrient Distribution

There was a significant positive association between the “healthy” pattern and percentage of protein contributing to total energy intake, and an inverse association between percentage protein intake and the “energy-dense meat” pattern. There were no significant associations with percentage protein intake between the “snacking” or the “fruit and vegetable” patterns. In addition, when comparing to the acceptable macronutrient distribution range (AMDR), mean percentage protein intake was within the AMDR (15%–25% of total energy intake) for all dietary pattern tertiles.

For the “healthy” pattern, there was a significant inverse association with percentage of carbohydrate intake. However, there were no significant associations between the “snacking”, “energy-dense meat”, or the “fruit and vegetable” patterns and percentage carbohydrate intake. Mean percentage carbohydrate intake was below the AMDR (45%–65% of total energy intake) for all dietary pattern tertiles. 

There was a significant positive association between the “healthy” pattern and percentage total fat intake, and an inverse association between the “fruit and vegetable” pattern and percentage of total fat intake. In addition, when comparing to the AMDR, mean percentage total fat intake was above the AMDR (20%–35% of total energy intake) for those with higher scores in the “energy-dense meat” and “healthy” patterns.

There was a significant positive association between the “energy-dense meat” pattern and percentage saturated fat intake, and energy intake, with an inverse association for percentage fiber intake. There was also an inverse association between the “fruit and vegetable” pattern and percentage saturated fat intake although a significant positive association existed for fiber intake. Mean percentage saturated fat intake was above the AMDR (<10% of total energy intake) for all dietary pattern tertiles (see Table 4). It is further clear that for all patterns except the “energy-dense meat” pattern, fiber intakes increase as the energy intakes increased across tertiles. However, for the “energy-dense meat” pattern the opposite was apparent; the higher the energy intake across tertiles, the lower the fiber intake. The highest energy intake in any tertile was in T3 of the “energy-dense meat” pattern whilst the highest fiber intake in any tertile was in T3 of the “fruit and vegetable” pattern.

## 4. Discussion

Four dietary patterns (“snacking”, “energy-dense meat”, “fruit and vegetable”, “healthy”) were identified in this group of NZE women. Older women were more likely to follow the “snacking” pattern, and younger women were more likely to follow the “energy-dense meat” pattern. After adjusting for energy intake, no dietary patterns were significantly associated with BMI or BF%. Evidence is mixed when investigating the association between dietary patterns and BMI, and two reviews concluded no clear associations [28,29]. Some studies have found an inverse association [30], a positive association [31,32], and others, no association between energy-dense patterns and BMI [33]. One review found dietary patterns in women to appear more weakly associated with BMI and obesity compared to men, indicating other factors may also play a part in determining women’s body composition [28,34]. Few studies, we are aware of, have investigated dietary patterns in relation to BF% [35]. When investigating macronutrient intakes, high and low consumers of all dietary patterns had carbohydrate intakes below and saturated fat intakes above the AMDR. The high saturated fat intakes are consistent with the NZ National Nutrition Survey, where intakes were above the AMDR for females [27]. The “snacking”, “energy-dense meat”, “fruit and vegetable”, and “healthy” patterns will be discussed in relation to our findings.

The “snacking” pattern was characterized by cakes and biscuits, sweet and savory snack foods (e.g., chocolate and potato chips) and peanut butter. This pattern is similar to the “junk food” pattern identified by Kourlaba et al. [36] and the “snacky” pattern identified by Aranceta et al. [37]. A significant difference in age was observed for the “snacking” pattern with older women more likely to follow this pattern (33.9 ± 7.9 years) compared with younger women (31.9 ± 8.7 years). This suggests that women who are older may eat more snack-like foods compared to younger women. McNaughton et al. [24] did not find a significant association between age and a dietary pattern characterized by chocolate, confectionary, added sugar, and fruit drink in women, however this pattern included additional foods to our snacking pattern (e.g., dairy milk and yoghurt). With an increasing availability of energy-dense food and the higher prevalence of overweight and obesity [1], the increased snacking in older pre-menopausal women (younger than 45 years) is plausible, as snacking has been shown to significantly contribute to excess energy consumption [9].

Women across all tertiles of the “snacking” pattern met the AMDR for percentage total energy intake from protein; however all dietary pattern tertiles were at the top end of the AMDR for total fat, above the AMDR for saturated fat (34.5% ± 5.6% and 13.4% ± 3.0% of total energy intake, respectively), and below the AMDR for carbohydrate (42.6% ± 7.3% of energy intake). Similarly, Pryer et al. [38] identified a “convenience” pattern followed by women who had the highest total fat intakes out of all patterns with 40.5% of energy coming from total fat. This shows that snack foods may contribute large amounts of fat to dietary intake. However, in the “snacking” pattern there were no significant differences in macronutrient intakes between the tertiles of this pattern, suggesting that the macronutrient intakes of women do not significantly change regardless of whether they follow the “snacking” pattern or not. 

The “energy-dense meat” pattern was categorized by red, white, and processed meat, puddings and crumbed and deep fried foods (e.g., crumbed and battered fish). This pattern is similar to the “Western” pattern identified by Schulze [14] or the “empty calorie” pattern identified by Quatromani et al. [39]. The “energy-dense meat” pattern had a significant positive association with BMI and an inverse association with age. However, after adjusting for energy intake, the association between the “energy-dense” meat pattern and BMI was no longer significant. Other studies [24,40] found younger individuals are more likely to follow the similar “Western” dietary pattern. Adolescents have been shown to have an increased consumption of soft drinks and fast-food, both of which are similar to this “energy-dense meat” dietary pattern [41]. The “Western” pattern has been associated with an increase in weight gain and a higher risk of being overweight [14,39], while Slattery [31] and Hu [32] found a “Western” dietary pattern to be associated with a higher BMI. Other studies, have found no association between dietary patterns and BMI [33,42]. These controversial findings may be explained by differences in study designs, study populations, and the dietary patterns identified. For example, Beaudry [42] included men and pre- and post-menopausal women, and Fung [33] included only male participants. In our study, BF% was significantly associated with the “energy-dense meat” pattern after controlling for age, but not energy intake. We are aware of only one other study to date which has investigated dietary patterns and BF% [35]. Tucker et al. [35] found a similar “Meat” dietary pattern which was associated with a significantly higher BF% which remained after adjusting for energy intake. This is similar to our findings and thus further investigation is warranted surrounding the influence of specific dietary patterns on BF% in premenopausal women.

Women with low scores on the “energy-dense meat” pattern had a higher percentage total energy intake from protein compared to those with higher scores. The “energy-dense meat” pattern is characterized by red, white, and processed meats, puddings and deep fried foods and high fat cheese, contributing to both protein and saturated fat. Increased red meat consumption has been associated with increased BMI due to its high energy and saturated fat content [43]. The high meat consumption likely contributes to the high saturated fat intakes seen in women following this “energy-dense meat” pattern. After adjusting for energy intake, no association was seen between the “energy-dense meat” pattern and BMI. Other studies found overweight and obese individuals are more likely to consume a greater amount of saturated fat compared to those of normal weight [44], and McCroy et al. [34] identified percentage of dietary fat to be positively associated with body fatness.

High intakes of all fruits and vegetables, including starchy vegetables but excluding potatoes comprised the “fruit and vegetable” pattern. This pattern is similar to the “healthy” pattern identified by Suliga [45] and Newby et al. [46], however both of their patterns included other items such as dairy and fruit juice. The “fruit and vegetable” dietary pattern was not significantly associated with age, BMI or BF%. Prudent patterns which also have high fruit and vegetable intakes have been associated with a lower BMI, however these patterns typically also have additional items such as legumes, wholegrains, fish, and poultry [32]. These additional food items may account for the lack of association seen between the “fruit and vegetable” pattern and BMI or BF%. Nevertheless, other studies suggest further research is required into which aspects of dietary patterns are preventative of increased BMI [46]. Additionally, the lack of association suggests additional factors may influence body composition in individuals following this pattern such as lack of physical activity, and socio-economic status, or dietary factors not accounted for using dietary pattern analysis. 

The majority of women who scored high on the “fruit and vegetable” pattern had mean ± SD total fat as a percentage of total energy intake lower (34.1% ± 7.3%) and within the AMDR guidelines, compared to the women who consumed only a few items from this pattern who had mean ± SD intakes exceeding the recommendations (36.8% ± 6.5%). Similarly, Schulze [14] also found individuals with lower fruit and vegetable intakes to have a diet higher in energy and total fat. On average, women less likely to follow the “fruit and vegetable” pattern had significantly higher total and saturated fat intakes and lower fiber intakes compared to those more likely to follow the pattern suggesting that more foods higher in both total and saturated fat may be consumed in the presence of lower fruit and vegetable consumption. Similarly, less fiber rich foods were consumed by those following the “energy dense meat” pattern, and the most was consumed by the highest consumers in the “fruit and vegetable” pattern. This was also observed by Tucker et al. [35], reporting that women with poor intakes of fruit, starch, fiber, and non-starchy vegetables had high intakes of meat, other carbohydrates (added sugars), and fat. As expected, we found, for all patterns except the “energy dense meat” pattern, that as energy intakes increased, fiber intakes also increased. 

The “healthy” pattern comprised fish and seafood, legumes, nuts, and seeds. This pattern was similar to the “bean” pattern identified by Maskarinec et al. [47]. The “healthy” pattern was not significantly associated with age, BMI or BF%, indicating that other factors; such as those suggested above, may play a role in determining body composition in individuals following this dietary pattern. 

Women scoring highly on the “healthy” pattern had significantly higher protein intakes (as a percentage of total energy intake) compared with women who scored low on this pattern. This pattern was associated with protein-rich items such as fish and seafood, legumes, eggs, and soy products which likely contribute to the higher protein content of their diet. Increases in protein intake, especially obtained from meat alternatives have been associated with increased satiety and weight regulation [48] as shown by Maskarincec et al. [47] who identified an inverse relationship between a “bean” pattern and BMI. Women following the “healthy” pattern also had significantly higher total fat intakes as a percentage of total energy intake in excess of the AMDR (36.7% ± 7.9%), compared to those less likely to follow this pattern. Despite the higher intake of fats, there was no association between the “healthy” dietary pattern and BMI levels. Foods rich in mono- and poly-unsaturated fatty acids (such as fish, nuts, and seeds) have previously been described as characterizing healthy dietary patterns, such as the Mediterranean diet, which are associated with a good metabolic profile due to its high content of such “healthy” fats [49]. It can be speculated that this may be due to the high mono- and poly-unsaturated fatty acid content of many of the foods associated with this pattern such as fish, nuts, and seeds. The “healthy” pattern also had significantly different carbohydrate intakes between the tertiles with women scoring high on this dietary pattern consuming less carbohydrate. The carbohydrate intakes in all tertiles of the “healthy” pattern were however below the AMDR and this trend; although not significant between the tertiles, was also present among the “snacking”, “energy-dense meat”, and “fruit and vegetable” patterns. 

Several factors may explain the lower carbohydrate intakes seen among participants in this study. Firstly, a low carbohydrate diet may be eaten to compensate for the consumption of foods with undesirable nutrient profiles. For example, consuming fewer carbohydrates means this energy needs to be replaced with protein or fat to maintain satiety and body weight [15]. The macronutrient distributions discernable from the women’s dietary patterns suggest that they compensate the low carbohydrate intakes with a higher consumption of foods higher in saturated fat. Secondly, a low carbohydrate diet has been highly publicized as a healthy diet to maintain or lose weight [50], with dietary trends currently promoting “high fat low carbohydrate” and “paleo” diets [51]. Individuals concerned about their health or weight often follow these trends and this may be why the macronutrient intakes in these women reflect this way of eating. Finally, social desirability and social approval bias have the potential to severely skew participant reporting [52]. Scagliusi et al. [53] found that women were more likely to report their energy intake in a socially desirable way and food items which are considered “fattening” or “unhealthy” such as confectionary, fried foods, and refined breads were more likely to be under reported. 

This study has several strengths. Firstly, the use of a homogenous group of women allows clear associations to be drawn between premenopausal women aged 16 to 45 years and the dietary patterns identified. This reduces variation and simplifies analysis by bringing focus to this particular group of women. We can be confident that these associations are relevant as other factors have been eliminated such as ethnicity. Secondly, the study addressed BMI and objectively measured BF% using ADP, which enabled both factors to be investigated in regard to their relationship with the dietary patterns. Investigating them separately enabled the relationship between body fatness and dietary patterns to be investigated as opposed to general BMI which may misclassify some individuals, especially those with normal weight obesity [54]. 

This study also has several limitations. Firstly, the study used retrospective reporting of dietary intake. It is difficult to assess dietary intake and measurement error can arise from a number of sources which may contribute to the lack of association between the dietary patterns and body composition [55]. Secondly, social desirability and social approval bias may have influenced the reporting of the NZE women with women reporting higher consumption of foods associated with the “fruit and vegetable” and “healthy” dietary patterns if they perceive these foods to be beneficial. This is a concern in all self-report dietary assessments [55]. In an attempt to reduce this bias, a validated FFQ was used which was shown to have good validity in ranking nutrient intake in relation to a food record over the last month of consumption [19]. Thirdly, this study did not adjust for confounding factors such as physical activity, socioeconomic status, and health behaviors such as smoking. This could have biased the analysis given the well-established relationship between physical activity and BMI and body fat mass [56]. In addition, the relatively small amount of total variance explained by the principal component factor analysis is also a limitation. The small amount of variance explained means that other dietary factors not captured by the dietary patterns may also influence the body composition (BMI and BF%) of these women. Finally, as this study involved volunteer participants, the study is not generalizable to all NZ women.

## 5. Conclusions

Factor analysis revealed four main dietary patterns followed by the NZE women. These were a “snacking”, “energy-dense meat”, “fruit and vegetable”, and “healthy” dietary pattern. Older women were most likely to follow the “snacking” pattern (characterized by cakes and biscuits, sweet and savory snack foods such as chocolate and potato chips, and peanut butter). Younger women are more likely to consume diets reflecting the “energy-dense meat” pattern (characterized by meat, refined grains, puddings, and fried foods) and these may promote adiposity through excess energy intake. Further research should be conducted to investigate the relationship between dietary patterns and body composition in pre-menopausal women when adjusting for physical activity, socioeconomic status, and health behaviours such as smoking. Additionally the high fat low carbohydrate intake of these NZE women warrants investigation into their cholesterol status and cardiovascular disease risk. 

## Figures and Tables

**Table 1:** Characteristics of all New Zealand European participants.

Characteristics	*n* (%)	NZE Women (*n* = 231)
Age at testing (years)		31.9 (25.2, 39.5) ^b^
16–24	54 (23.4)	
25–35	88 (38.1)	
36–45	89 (38.5)	
Height (cm)		167 ± 6.6 ^a^
Weight (kg)		70.1 ± 14.2 ^a^
Body Mass Index (BMI) (kg/m^2^) ^c^		24 (21.7, 27.2) ^b^
Normal (18.5–24.99)	140 (60.6)	
Overweight (25.0–29.99)	60 (26)	
Obese (≥30.0)	31 (13.4)	
Body fat percentage (%)		32.6 ± 8.1 ^a^
Under (<22)	18 (7.8)	
Normal (22–29.9)	66 (28.6)	
High (≥30)	147 (63.6)	
Waist circumference (cm)(<80) ^c^		78.1 ± 11.0 ^a^
Hip circumference (cm)		104.2 ± 10.6 ^a^
Waist: Hip ratio (<0.85) ^c^		0.7 ± 0.1 ^a^

^a^ = Mean ± Standard deviation; ^b^ = Median (25th, 75th percentile); ^c^ = Standard reference values [3,25].

**Table 2:** Factor loading matrix for the four dietary patterns identified in NZE women (*n* = 231), aged 16–45 years.

	Dietary Patterns			
	P1 Snacking	P2 Energy-Dense Meat	P3 Fruit and Vegetable	P4 Healthy
Full fat milk	-	-	-	-
Low fat milk	-	-	-	-
High calcium milk	0.284	−0.238	-	-
Sweetened milk products	-	0.297	-	-
Yoghurt	-	−0.214	-	-
High fat cheese	-	0.361	-	-
Low fat cheese	0.303	-	-	-
Apple, Banana, Orange	-	-	0.424	-
Other fruit	-	-	0.568	-
Tomatoes	-	-	0.476	-
Dark-yellow vegetables	-	-	0.628	-
Green vegetables	−0.249	-	0.588	-
Other non-starchy vegetables	-	-	0.673	-
Potatoes	-	0.402	-	-
Starchy vegetables	-	-	0.593	-
White breads	-	0.480	-	-
Discretionary breads		0.422	-	-
Crackers	0.280	-	-	-
Brown breads	0.384	-	-	−0.225
Refined grains	-	0.390	-	-
Wholegrain	-	-	-	0.583
Non sweetened cereals	-	-	-	-
Sweetened cereals	-	-	-	−0.291
Red meats	−0.231	0.609	-	-
White meats	-	0.495	-	-
Processed meats	-	0.577	-	-
Fish and seafood	-	-	-	0.502
Egg and egg dishes	−0.233	-	-	0.293
Legumes	-	-	-	0.546
Soy products	-	−0.248	-	0.398
Peanut butter and peanuts	0.549	-	-	-
Nuts and seeds	-	-	-	0.411
Coconut fats	-	-	-	0.462
Margarine	0.538	-	-	-
Creamy dressings	-	0.297	-	-
Sauces	-	0.396	-	-
Sweet spreads	0.572	-	-	-
Savory spreads	0.336	-	-	-
Cakes and biscuits	0.674	-	-	-
Pudding	-	0.565	-	-
Sweet snack foods	0.477	-	-	-
Savory snack foods	0.531	-	-	-
Crumbed and deep fried	-	0.544	-	-
Fast food	-	-	-	-
Fruit juice	-	-	-	-
Fruit and other drinks	-	0.328	-	-
Tea	0.206	-	-	-
Coffee	-	-	−0.245	-
Beer	-	-	-	0.234
Wine	-	-	-	-
Water	-	−0.279	-	0.430
Other alcoholic beverages	-	0.312	-	-
Explanation of variation of food intake (%)	6.9	6.8	5.6	4.8
Final Cronbach’s alpha ^#^	0.639	0.634	0.648	0.431

P1, P2, P3, P4 = four dietary patterns, factors identified based on factor loadings >0.2; Cross-loadings between patterns were removed; Food groups with loadings <0.2 are not shown. Cronbach’s α values indicated a moderate inter-item reliability; ^#^ Final Cronbach’s α values improved following removal of white sugar, and diet drinks in pattern 1, oats in pattern 2, and oil-based dressings, fats and soy milk in P4; KMO = 0.613; Bartlett’s test of sphericity = 0.000.

**Table 3:** Associations between dietary patterns and age, body mass index (BMI) and body fat percentage (BF%) in NZE women, aged 16–45 years (*n* = 231).

Patterns	Age (Years)	*p*-Value	BMI (kg/m^2^)	*p*-Value	BF%	*p*-Value
P1: Snacking						
T1	31.9 ± 8.7 *	0.011 ^a^	24.2 ± 3.7		31.9 ± 7.3	
T2	29.9 ± 8.0 ^c^		25.2 ± 4.8		32.1 ± 8.4	
T3	33.9 ± 7.9 ^c^		25.9 ± 6.5		33.7 ± 8.5	
P2: Energy-dense meat				0.016 ^b^		
T1	32.8 ± 8.2 *^,e^	0.012 ^a^	24.6 ± 4.2 *^,e^		32.2 ± 7.4	0.016 ^b^
T2	32.9 ± 8.1 ^d^		24.4 ± 4.0 ^d^		31.4 ± 7.2	
T3	30.0 ± 8.3 ^d,e^		26.4 ± 6.7 ^d,e^		34.3 ± 9.2	
P3: Fruit and vegetable						
T1	31.7 ± 7.8		24.8 ± 4.4		33.2 ± 7.2	
T2	31.1 ± 8.4		24.4 ± 4.3		31.5 ± 7.2	
T3	32.9 ± 8.6		26.2 ± 6.4		33.1 ± 9.5	
P4: Healthy						
T1	32.2 ± 8.6		25.2 ± 4.8		33.7 ± 7.5	
T2	31.6 ± 8.6		25.5 ± 5.6		33.0 ± 8.1	
T3	31.9 ± 7.6		24.8 ± 5.1		31.1 ± 8.4	

Values are means ± standard deviations; T1, T2, T3 = tertiles of dietary pattern score; * *p* <0.05 following ANOVA analysis within each pattern, between tertiles; ^a^ = energy controlled for; ^b^ = age controlled for, ^c–e^ = values with the same superscript letters are significantly different according to both Tukey HSD and Gabriel post hoc tests.

**Table 4:** Macronutrient distribution profile of dietary patterns in NZE women aged 16–45 years (*n* = 231).

Macronutrient	Snacking Pattern			Energy-Dense Meat Pattern			Fruit and Vegetable Pattern			Healthy Pattern		
	T1	T2	T3	T1	T2	T3	T1	T2	T3	T1	T2	T3
	(% of EI)			(% of EI)			(% of EI)			(% of EI)		
Energy (kJ)	8516 ± 2793	9100 ± 2862	9420 ± 3204	7120 ± 2161	8758 ± 1982	11179 ± 3095 *	8425 ± 3040	8833 ± 2617	9761 ± 3103 *	9138 ± 3368	8473 ± 2950	9450 ± 2462
Protein (15%–25%) ^a^	18.5 ± 3.0	18.3 ± 3.3	17.8 ± 3.8	19.1 ± 3.8	17.9 ± 3.4	17.8 ± 2.9 *	17.9 ± 3.7	18.3 ± 2.9	18.6 ± 3.7	17.6 ± 2.9	18.1 ± 3.3	19.2 ± 3.9 *
Carbohydrate (45%–65%) ^a^	40.2 ± 7.8	42.1 ± 6.7	42.6 ± 7.3	41.5 ± 7.1	41.2 ± 8.5	41.9 ± 7.0	40.1 ± 8.1	42.1 ± 6.2	42.3 ± 8.1	43.2 ± 5.8	42.5 ± 6.4	38.7 ± 9.3 *
Fat (20%–35%) ^a^	35.9 ± 7.6	34.5 ± 5.9	34.5 ± 5.6	33.8 ± 6.3	35.3 ± 6.9	35.9 ± 6.3	36.8 ± 6.5	34.2 ± 5.3	34.1 ± 7.3 *	34.5 ± 5.6	33.8 ± 5.4	36.7 ± 7.9 *
Saturated fat (<10%) ^a^	14.6 ± 4.6	13.6 ± 2.9	13.4 ± 3.0	12.5 ± 3.0	14.1 ± 3.4	15.0 ± 3.9 *	14.8 ± 3.7	13.7 ± 2.8	13.1 ± 4.1 *	14.3 ± 3.6	13.3 ± 2.7	13.9 ± 4.4
Fiber	2.6 ± 0.7	2.7 ± 0.7	2.8 ± 0.8	3.1 ± 0.8	2.6 ± 0.6	2.3 ± 0.6 *	2.3 ± 0.6	2.7 ± 0.6	3.1 ± 0.7 *	2.4 ± 0.7	2.8 ± 0.8	2.9 ± 0.7 *

Values are means ± standard deviation; * *p* < 0.05 following ANOVA analysis within each pattern, between tertiles; T1, T2, T3 = tertiles of dietary pattern score; EI = Energy intake; ^a^ = Macronutrient distribution range [27].

## Supplementary Materials

The following are available online at http://www.mdpi.com/2072-6643/8/8/450/s1, Table S1: Food groups.

## Abbreviations

The following abbreviations are used in this manuscript:

ADP	Air displacement plethysmography
BF%	Body fat percentage
BIA	Bioelectrical impedance analysis
AMDR	Acceptable macronutrient distribution range
ANOVA	Analysis of Variance
ANCOVA	Analysis of Covariance
BMI	Body mass index
EI	Energy intake
EXPLORE	Examining the Predictors Linking Obesity Related Elements
NZWFFQ	New Zealand women’s food frequency questionnaire
GI	Glycemic index
HNRU	Human Nutrition Research Unit
KMO	Kaiser-Meyer-Olkin
N	Number
NZ	New Zealand
NZE	New Zealand European
P1	Pattern 1
P2	Pattern 2
P3	Pattern 3
P4	Pattern 4
SD	Standard deviation
T1	Tertile 1
T2	Tertile 2
T3	Tertile 3
W	Week
