# Supplementary material for: Exploring the Dietary Patterns of Young New Zealand Women and Associations with BMI and Body Fat
Source: Nutrients. 2016 Jul 26;8(8):450. doi: 10.3390/nu8080450 (PMC4997365; doi:10.3390/nu8080450)
Supplement: Supplementary file 1 [file nutrients-08-00450-s001.docx]

Supplementary Materials: Exploring the Dietary Patterns of Young New Zealand Women and Associations with BMI and Body Fat

Jenna K. Schrijvers, Sarah A. McNaughton, Kathryn L. Beck and Rozanne Kruger

**Table S1.** Food groups.

| **Food Group** | **Food Items Included** |
| --- | --- |
| Full fat milk | Full fat milk (dark blue top) |
| Low fat milk | Lite milk (light blue top), trim milk (green top) |
| Sweetened milk products | Breakfast drinks, flavored milk, evaporated milk, hot chocolate |
| Yoghurt | Yoghurt (plain, fruity, Greek, unsweetened) |
| High fat cheese | Cheddar, processed cheese, cream cheese, blue vein |
| Low fat cheese | Edam, cottage cheese, brie, camembert, feta |
| Apple, banana, orange | Apple, banana, orange |
| Other fruit | Fresh, canned, frozen, dried |
| Tomatoes | Tomatoes |
| Dark-yellow vegetables | Carrots, pumpkin |
| Green vegetables | Lettuce, spinach, cabbage, broccoli, watercress, green beans, sprouts, courgette |
| Other non-starchy vegetables | Capsicum, onion, mushrooms, frozen mixed vegetables, beetroot |
| Potatoes (excluding chips and crisps) | Potato (boiled, mashed, baked, stuffed, scalloped) |
| Starchy vegetables | Kumara, yam, parsnip, turnip, swedes (boiled, mashed, baked), Taro (flesh, roots, stalks), green banana, sweet corn kernels |
| White breads | White bread, wraps, fruit bread, focaccia, bagel, pita, paraoa bread, rewena bread, doughboys |
| Discretionary breads | Crumpets, scone, savory muffin, croissant, pancakes/waffles, iced bun |
| Crackers | Cream, cruskit, corn, rice, vitawheat |
| Whole grain breads | Bread (high fiber, wholemeal, wholegrain) |
| Refined grains | White rice, pasta (penne, spaghetti, vermicelli), noodles (instant, egg, rice), canned spaghetti |
| Wholegrains | Brown rice, quinoa, couscous, bulgur wheat |
| Oats | Porridge, rolled oats, oat bran, oat meal |
| Sweetened cereals | Milo cereal, coco pops, nutrigrain, honey puffs, fruit loops, special K, light and tasty, sultana bran |
| Red meats | Beef (mince dishes, casserole, stew, stir-fry, roast, steak), Lamb (stew, casserole, stir-fry), Venison, hogget (roast, chops, steak, casserole, stew, stir-fry), offal (liver, kidney, pate) |
| White meats | All chicken (breast, leg, wing, casserole, stir-fry), Turkey/quail, pork (roast, chop, steak), mutton bird/duck, veal |
| Processed meats | Sausages, frankfurters, saveloys, cocktail sausages, bacon, ham, luncheon meats, salami, chorizo, meatloaf, corned beef, patties |
| Fish and seafood | Canned salmon, canned tuna, canned mackerel, Snapper/hoki, gurnard, shark, tuna, salmon, Shrimp/prawn, crab, mussels, pipi, whitebait, kina, squid |
| Egg and egg dishes | Eggs, egg mixed dishes (omelette, quiche, frittata, other baked egg dishes) |
| Legumes | Canned/dried (lentils, chickpeas, peas, beans, baked beans), hummus, dahl |
| Soy products | Soybeans, tofu |
| Peanut butter and peanuts | Peanut butter, peanuts |
| Nuts and seeds | Nuts (peanut, brazil, walnut, almond, cashew, pistachio), Seeds (pumpkin, sunflower) |
| Fats | Butter, lard, dripping, ghee |
| Coconut fats | Coconut milk, cream, oil |
| Oil and oil-based dressings | Canola, sunflower, olive, vegetable oils, cooking sprays, Salad dressing (French, Italian), Avocado |
| Margarine | Margarine—all types |
| Creamy dressings | Mayonnaise, creamy dressings, white/cheese sauce, sour cream |
| Sauces | Tomato, barbeque, chilli, mint, soy, gravy, mustard, chutney, instant soup |
| Sweet spreads | Jam, honey, marmalade |
| Savory spreads | Vegemite, marmite |
| Cakes and biscuits | Cakes, loaves, muffins, sweet pies, pastries, tarts, doughnuts, biscuits (plain, chocolate coated) |
| Puddings | Ice cream, custard, milk puddings (semolina, instant), Other non-dairy based puddings (pavlova, sticky date pudding), jelly, ice blocks |
| Sweet snack foods | Chocolate, candy/confectionary, muesli bars |
| Savory snack foods | Potato chips, corn chips, twisties |
| Crumbed and deep fried | Crumbed chicken/fish, battered fish, potato fries, chicken nuggets |
| Fast-food | Meat pie, sausage roll, savories, burgers, kebab, Chinese, Indian, Thai, Pizza |
| Fruit juice | Fruit and vegetable juice |
| Fruit drink and other beverages | Fruit drink, sparkling grape juice, cordial, iced tea, energy drinks, sports drinks, flavored water, soft drinks |
| Diet drinks | Diet energy drinks, diet soft drinks, diet cordial |
| Tea | Black tea, herbal tea |
| Coffee | Instant coffee, brewed water-based coffee, espresso |
| Beer | Standard, low alcohol |
| Wine (red and white) | White wine, red wine |
| Water | Water |
| Other alcoholic beverages | Cider, spirits, sherry, port, ready-to-drink alcoholic sodas (RTD’s), kava |
| Sugar added to food and drink | White sugar |
